# Stochastic techno-economic analysis of the production of aviation biofuel from oilseeds

**DOI:** 10.1186/s13068-018-1158-0

**Published:** 2018-06-08

**Authors:** Ana Paula M. M. Diniz, Richard Sargeant, Graeme J. Millar

**Affiliations:** Institute for Future Environments, Science and Engineering Faculty, Queensland University of Technology (QUT), Brisbane, QLD Australia

**Keywords:** Aviation biofuel, Sustainable aviation fuel, Conversion technologies, Oilseeds, Techno-economic analysis, Stochastic models

## Abstract

**Background:**

The economic viability of hydrodeoxygenation process using Camelina, Carinata and Jatropha feedstocks for aviation biofuel production was evaluated for two product profiles: (i) maximum diesel production and (ii) maximum jet fuel production (HRJ).

**Results:**

Deterministic analysis of Camelina and Carinata diesel facilities returned positive NPVs and IRRs of 25 and 18%, respectively. Stochastic analysis suggested that the probabilities of positive NPVs were 75, 59 and 15%, respectively, for Camelina, Carinata and Jatropha diesel plants. Jet fuel facilities presented probabilities of loss of 98, 99 and 100% for Camelina, Carinata and Jatropha scenarios, respectively. Sensitivity analysis determined that financial performance was majorly influenced by feedstock and fuel prices. Categories of subsidies to enhance the attractiveness of the projects were studied.

**Conclusions:**

Camelina, Carinata and Jatropha plants targeting HRJ required incentives of 0.31, 0.39 and 0.61 US$/L of biofuel produced, respectively, to reduce the probabilities of loss to approximately 30%.

**Electronic supplementary material:**

The online version of this article (10.1186/s13068-018-1158-0) contains supplementary material, which is available to authorized users.

## Background

It is widely accepted that human activities are responsible for global warming [1], with the US Environmental Protection Agency [2] stating that the largest source of greenhouse gas emissions is attributed to the combustion of fossil fuels. Bittner et al. [3] reported that the transportation sector is responsible for 32% of the total carbon dioxide emissions in the world. Consequently, the replacement of petroleum-derived transportation fuels by renewable alternatives is a crucial step in mitigating the greenhouse effect [4].

The aviation industry accounts for a significant fraction of global transportation needs [4]. Both Rodrigue et al. [5] and Klein-Marcuschamer et al. [4] noted that in 2010, air transport was responsible for about 10% of the transportation sector’s energy use. Bittner et al. [3] estimated that this latter share of energy use would increase to 13% by 2040. Regarding greenhouse gas emissions, the aeronautic sector is one of the most rapidly growing sectors, with a growth rate of 87% since the early 1990s [6]. Presently, it is responsible for 2% of the world’s anthropogenic carbon dioxide emissions [3], and due to the projected increase in demand it is expected to be the most polluting transportation segment by 2050 [6]. Advances in air traffic management and technological improvements to enhance engine efficiency have the potential to reduce emissions, but these measures are considered to be insufficient to compensate for the increase in passenger numbers [7]. Therefore, emission reductions from the aviation sector will require adoption of cleaner fuel alternatives.

The most widely used aviation fuels are JET A-1, a kerosene-type fuel used in gas turbine-powered aircrafts, and AVGAS, a gasoline-type fuel used in small piston engine-powered aircrafts [6]. Several authors are in agreement that the replacement of current aviation fuels cannot be met by alternatives such as battery-powered engines or compressed natural gas-powered engines [3, 4, 8]. Instead, the development of jet fuels derived from renewable energy sources which are competitive with petroleum fuels in terms of efficiency and price may be the preferred alternative to decrease emissions from the aviation sector. According to Chu et al. [9], the use of renewable feedstocks could provide aviation with 10–50% reduction in emissions. Besides these concerns about the environment, renewable fuels would also reduce the dependency on fossil fuels, which are limited in supply and being depleted at a rapid rate [10].

There are several types of feedstocks which are potentially amenable to synthesis of aviation biofuel. However, feedstocks derived from food crops, such as corn and soybean, are contentious as they may be better used as food for humans and animals [11]. SkyNRG, established to be a global market leader for sustainable jet fuel, agrees that most of the sustainability impacts of biofuels are directly related to the feedstock [12]. In response to these concerns, the aviation industry is now committed to use only second-generation feedstocks, which: do not compromise food security; use minimal land area; require relatively low water and energy resources; minimize impacts on biodiversity; and also provide socioeconomic value to local communities where biomass is grown. Oilseeds (Jatropha, Camelina, Carinata, macauba, babassu), waste biomass (used cooking oil), tallow (animal fat) and algae are some examples of these feedstocks [13]. The precise feedstock choice should be evaluated case by case, since some of them can only be grown in specific regions of the world [14].

Biofuels have already been tested and utilized in commercial and military flights across the world. In Australia, Qantas operated the first commercial flight powered by sustainable aviation fuel in April 2012 [12]. The Airbus A330 was partly powered by biofuel derived from used cooking oil [15]. In accordance with a statement from SkyNRG, to be certified for commercial use, sustainable jet fuels have to meet strict specifications established by the American Society for Testing and Materials (ASTM) [14]. The certification is to guarantee safety and performance, and the specifications are related to flash point, freezing point, combustion heat, viscosity, sulphur content and density [14]. In addition to the final products, the ASTM also certifies the processes used to produce aviation biofuels [16].

Wang et al. [16] described several process technologies which can be used to convert biomass-based materials into alternative jet fuels. These latter technologies were noted to be dependent upon the type of feedstock, and some have already reached commercial demonstration scale, whereas others are still in the research and development stage. Five technologies for production of sustainable aviation fuel are of particular interest: Fisher–Tropsch (FT); alcohol to jet (ATJ); pyrolysis; direct sugars to hydrocarbons (DSHC); and from hydroprocessed esters and fatty acids (HEFA) [14]. Bio-jet fuels produced from FT, ATJ, DSHC and HEFA processes are presently approved for blending into current jet fuels at levels up to 50%. Mawhood et al. [7] further noted that the ASTM has formed a task force to work towards the certification of two other conversion technologies: hydrotreated depolymerized cellulosic jet (HDCJ) and aqueous phase reforming (APR). Nonetheless, the only conversion pathway ready for large-scale deployment are the hydroprocessing technologies using vegetable and waste oils [16].

Uncertainties related to the economic viability of production pathways for aviation biofuels have prevented investments in this sector. For Wang et al. [16], production cost was considered the key parameter for the commercial viability of a bio-jet fuel. Mawhood et al. [7] proposed that the potential to scale up renewable jet fuel volumes was severely restricted by the lack of low cost and sustainable feedstocks. Stelle et al. [11] considered that 85% of biofuel production costs were related to the cost of feedstocks, and production was the second major component of the total cost of the fuel. Bittner et al. [3] concluded that there were five major areas of uncertainty impeding investments in the biofuel industry: crude oil price; feedstock availability and cost; conversion technology yields and costs; environmental impacts; and government policy. In summary, to be economically viable, aviation biofuel must be cost competitive with fossil jet fuel. The global average price paid at the refinery for aviation fuel in August 2017 was $1.52/gal [17]. The predictions of both Stelle et al. [11] and Wang et al. [16] suggest that traditional jet fuel will become more expensive, as a consequence of the trends in petroleum prices, while sustainable aviation fuel will become cheaper as the industry develops.

Even though the biofuel industry is being largely incentivized by the government of several countries, mainly due to environmental concerns [18], it is mainly owned and operated by the private sector [3]. It is unlikely that private investors will provide substantial financing to support sustainable aviation fuel production projects without robust cost predictions [19]. According to Klein-Marcuschamer et al. [4], at the same time that there are many technologies being investigated, there are also a number of controversial and contradictory claims related to the performance of technologies and feedstocks or the advantages of some production routes over others.

Crawford et al. [20] affirmed that the combination of engineering and economics approach is critical for any technology to transition from research to industry. This means that a comprehensive techno-economic analysis to investigate the economic potential of different scenarios is an essential step for proceeding with the sustainability programme of the aviation industry. Techno-economic analyses of different production pathways and feedstocks for renewable jet fuels using a deterministic approach have been reported [4, 20–27]. However, there is a gap in establishing stochastic models capable of evaluating the extent to which uncertainties can influence the financial metrics [3, 9, 28–31]. In comparison with deterministic models, stochastic versions are considered to be more reliable, as they inherently assess risk. Instead of forecasting only the most likely rate of a variable, the projections of a stochastic model show a range of possible outcomes and how likely each outcome uses Monte Carlo simulation [32]. Chu et al. [9], for example, developed a techno-economic analysis of the production of bio-jet fuel from waste biomass and oil crops. Although some scenarios have been demonstrated to be economically viable under a deterministic approach, a stochastic assessment revealed that the probabilities of them achieving positive net present values ranged from 8 to 29% depending on the feedstock, indicating risky investments. This kind of information is far more valuable for decision makers because they contemplate prices volatility instead of using single point estimates, which makes the results more realistic. In addition, both Chu et al. and Bann et al. [9, 28] used Monte Carlo simulations to estimate policy supports that would enhance the attractiveness of the projects.

Therefore, the aim of this project was to examine the economic viability of the production of aviation biofuel, by comparing and contrasting both deterministic and stochastic approaches. The central hypothesis was that stochastic models may provide improved economic analysis which could accelerate the development of the aviation biofuel sector. The HEFA process, also known as hydrodeoxygenation (HDO) process, for converting Camelina, Carinata and Jatropha was considered. The selection of the process technology was made based on its certification and development stage [9, 16], and the selection of the feedstocks was made based on their characteristics and suitability to Australian agriculture conditions [10, 33–36]. Camelina, Carinata and Jatropha are known to have high oil content, be able to grow on arid lands and have high tolerance to heat, drought and pests.

The key research questions of the project were: (1) Is the production of aviation biofuel from the conversion process and oilseeds being analysed economically viable from a deterministic perspective? (2) What is the likelihood of these projects being economically viable when taking into consideration the volatility of the market? (3) What policies could be put in place to increase the attractiveness of these projects?

## Methods

### Process description

**Fig. 1:**
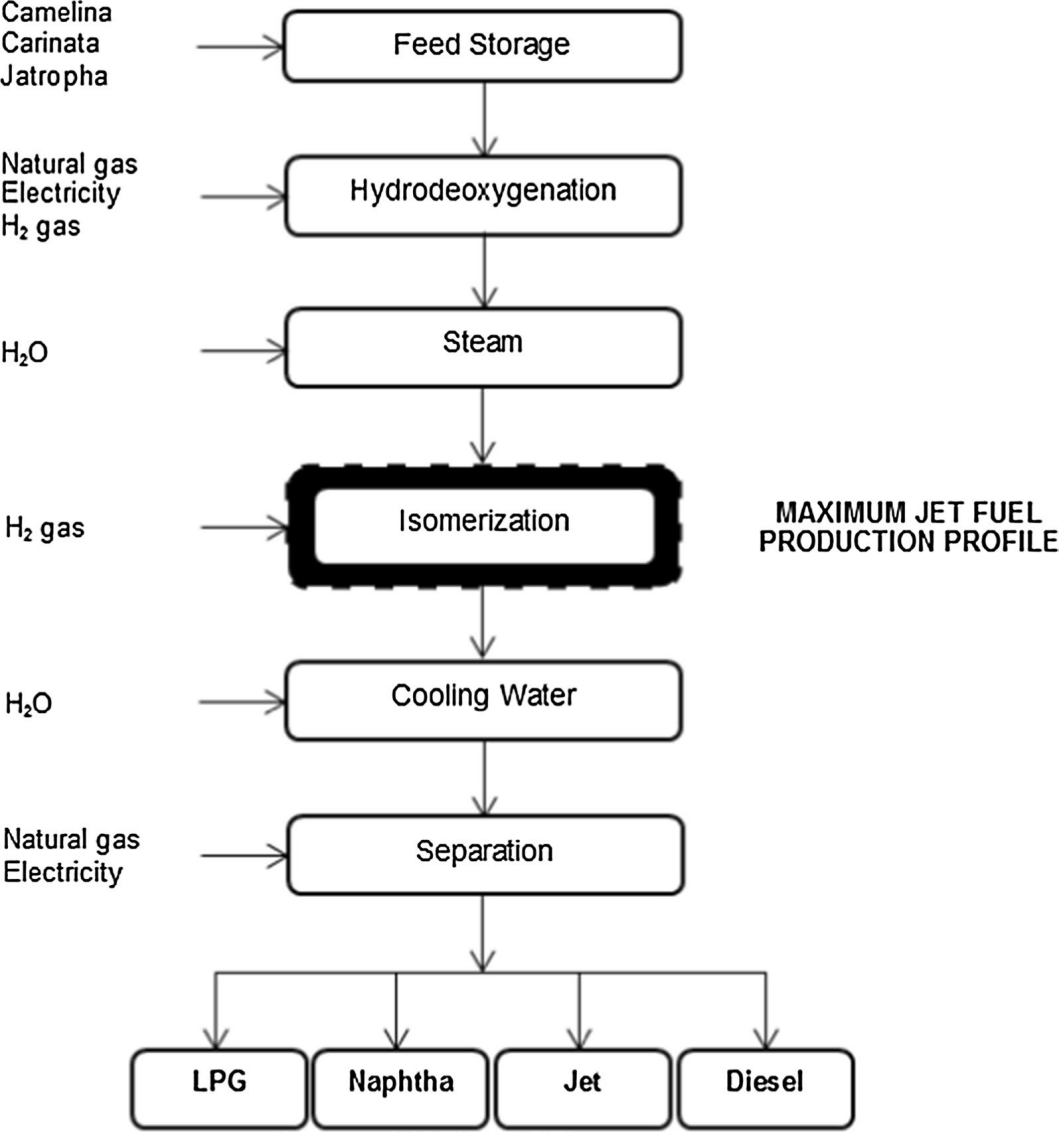
Simplified hydrodeoxygenation process design

Process technologies to produce sustainable jet fuels are dependent upon the type of feedstock [16]. Oil-based feedstocks are converted into bio-jet fuels through hydroprocessing technologies, which are the only conversion pathways considered to be ready for large-scale deployment as approved by ASTM. Pearlson et al. [23] reported that a life cycle greenhouse gas emissions assessment of the HDO process suggested a 60% reduction relative to conventional jet fuel technologies. These authors further added that this process had proved itself to be effective at producing drop-in quality fuels, which also brings significant strategic and financial benefits, since drop-ins are synthetic equivalents of petroleum products. The HDO process may be altered to create two different product profiles: maximum diesel production (HRD) and maximum jet fuel production (HRJ) [9, 24]. Maximizing the production of jet fuel demands additional processing (isomerization). However, some production of diesel is unavoidable. In the same way, when maximizing the production of diesel, some jet fuel will also be produced. An overview of the process is presented in Fig. 1.

Raw material is taken from feed storage and sent to a hydrotreater along with hydrogen gas. The deoxygenated effluent is cooled by steam generation, and, if maximizing the production of renewable jet fuel, sent to an isomerization unit. The isomerized hydrocarbon product is then cooled with cooling water before being sent to a separator where mixed paraffin gases, carbon dioxide and excess hydrogen are separated from the liquid products. Liquid products are separated into liquefied petroleum gas (LPG), naphtha, hydroprocessed renewable jet fuel and hydroprocessed renewable diesel.

### Evaluated scenarios

To assess the economic viability of the production of aviation biofuel, base case scenarios considering bio-jet fuel produced from Camelina, Carinata and Jatropha through both product profiles of the HDO process (HRD and HRJ) were studied. Information about facility designs, processes, utility requirements, product yields and financial data was extracted from previous literature [9, 21–24, 37, 38].

### Financial modelling

For this study, a weighted average cost of capital (WACC) model was the adopted method for calculating the appropriate discount rate used for determining the present value of future cash flows. This method estimates risks and returns for investments using a combination of firm-level and macroeconomic analysis, in which each category of capital is proportionally weighted [39, 40] (see Additional file 1: C for relevant equations).

Baseline cases were created using the assumptions summarized in Table 1, and the inputs and outputs that are described in the following sections. Data were taken from existing literature [9, 22–24, 39, 41] and transferred to an Australian context by including in calculations: the national historical inflation rate [42]; the income tax rate applied to small business entities [43]; and Australian prices for consumed utilities [44–46] and generated co-products [17, 47–52].

**Table 1:** Assumptions adopted in the financial analysis

	Value	Units
Plant life	20	Years
Annual operating hours	8400	h
Fixed feed rate	39	Tonnes of oil/h
Feedstock oil content		
Camelina	35	%
Carinata	44	%
Jatropha	33	%
Annual liquid fuel throughput		
HRD	382	Million litres per year (MLPY)
HRJ	398	Million litres per year (MLPY)
Depreciation	Straight line	
Salvage value	20	% of total capital investment (TCI)
Price escalation	2	%
Inflation rate	2	%
Debit interest rate	8	%
Income tax rate	29	%
Debt financing	50	% of total project investment (TPI)
Risk-free rate of interest	2	%
Expected return on the market portfolio	15	%
Stock rate of return	8	%
Market rate of return	9	%

Net present value (NPV) was calculated for each scenario. Internal rate of return (IRR) and payback time were also calculated when applicable. Break-even analyses were performed to estimate the minimum selling prices of the fuel products and the maximum cost of the feedstocks that set the NPV of the systems to zero. Variations and uncertainties related to the capital investment, oil content and prices of feedstocks, prices of utilities and prices of co-products were assessed and incorporated into stochastic models to predict the impact on the NPV of the facilities. These models were evaluated using the Palisade Decision Tools suite software package which incorporates @Risk and uses Monte Carlo simulations to account for the variance in the techno-economic parameters. Finally, a policy analysis was conducted to determine the magnitude of incentives that would be required to reduce the riskiness of the projects to limits that are considered acceptable by investors. All financial data are presented in 2017 US$. Prices in Australian dollars (e.g. natural gas, electricity, water) were converted to US dollars.

#### Product yields

The product quantities summarized in Table 2 are based on reported literature values [22–24, 37]. Hydrogen gas consumption and co-product distributions vary depending on the feedstock being used in the process [24]. However, due to the lack of information on the HRD product profile with regard to the feedstocks considered in this project, mass-based product yields were generalized taking into account values reported by Pearlson et al. [23, 24].

**Table 2:** Mass-based product yields by product profile and feedstock

	HRD			HRJ		
	Camelina	Carinata	Jatropha	Camelina	Carinata	Jatropha
Inputs						
Vegetable oil	1000	1000	1000	1000	1000	1000
Hydrogen gas	27	27	27	30	26	25
Outputs						
CO_2_	55	55	55	101	95	N/R
CO	N/R	N/R	N/R	2.7	3	N/R
Water	87	87	87	36	34	N/R
LPG	58	58	58	88	79	78
Naphtha	18	18	18	127	145	57
Kerosene	128	128	128	535	537	740
Diesel	681	681	681	140	132	107

Values expressed in kg per tonne of oil

*N/R* not reported

#### Utility requirements

The utilities demanded in the process are the same for both product profiles [24]. They include thermal energy (natural gas), electricity and water. The required amount of a particular utility, however, differs according to the feedstock, and appropriate values were taken from relevant literature [24, 37]. Table 3 outlines the utility requirements per tonne of each vegetable oil being analysed.

**Table 3:** Utility requirements per tonne of vegetable oil

	Thermal energy (GJ/tonne of oil)	Electricity (kWh/tonne of oil)	Water (kL/h/tonne of oil)
Camelina	6	227	5
Carinata	5	180	5
Jatropha	11	44	5

#### Capital expenses (CAPEX)

The CAPEX is the total cost to build the facility. It includes site preparation, excavation, plant construction, piping, electrical, instrumentation and control, equipment purchase and installation, commissioning costs and project contingency. Cost estimates for both analysed product profiles were obtained from the literature [9] and adjusted to 2017 US$ using the inflation rates in Australia [42]. More details are presented in Additional file 1: A.

The total equipment installed cost (TEIC) is the cost of the equipment and installation. The total direct cost (TDC) is the sum of the TEIC and the costs associated with site development. The total capital investment (TCI) is the sum of direct and indirect costs. The working capital (WC) is assumed to be 10% of the TCI. The total project investment (TPI) combined both the TCI and WC. The TPI for an HRD plant processing oilseeds was estimated to be US$253 MM, while the TPI for an HRJ facility was estimated at US$422 MM.

#### Operating expenses (OPEX)

The OPEX is the cost to run the plant and is divided into fixed and variable. Fixed operating expenses are constant and independent of production levels. Variable operating expenses are not constant and proportional to the level of production [24].

##### Fixed operating expenses

Fixed OPEX includes labour, overhead, maintenance, insurance, taxes and contingency. The assumptions used to calculate the facilities’ annual fixed operating expenses were derived from reported literature [9, 24] and are shown in Table 4.

**Table 4:** Fixed and variable operating expenses

Fixed expenses		Variable operating expenses		Unit prices (US$)
Labour	12 staff @ $72 k/year	Vegetable oil		
		Camelina		323/tonne seed
		Carinata		356/tonne seed
Overhead	0.2% of TPI	Jatropha		254/tonne seed
Maintenance	5.5% of TPI	Natural gas		3.05/GJ
Insurance	0.5% of TPI	Electric power		0.21/kWh
Taxes	1% of TPI	Water		1.62/kL
Contingency	10% of the above subtotal	Hydrogen gas		1.21/kg

Pearlson [24] conducted interviews with industry professionals who suggested that a reduced number of staff was required for biofuel plants, since the plant is not as complex as a conventional refinery, and an average annual salary of US$72,000 was assumed. Overhead costs are minor, representing an average value of 0.2% of the TPI. Maintenance includes costs for materials and labour and was considered to be 5.5% of the TPI. Insurance and taxes accounted for 0.5 and 1% of the TPI, respectively. A 10% contingency was included to manage uncertainties. The fixed OPEX for an HRD plant was estimated to be US$21 MM, whereas the fixed OPEX for an HRJ plant was approximately US$34 MM.

##### Variable operating expenses

Variable OPEX includes raw material, thermal energy, electricity, water and hydrogen gas. The unit prices used to calculate the plants’ total variable OPEX are presented in Table 4. Prices for Camelina, Carinata and Jatropha were based on the studies of Chu et al. [9] and Wang [22], and adjusted to 2017 US$. These prices included costs of seeding, fertilizer, herbicides, harvesting, machinery, labour, insurance, land and transportation. Utilities’ prices were taken from [9, 44–46] and all prices in Australian dollars were converted to US dollars.

#### Gross income

Hydroprocessing plants convert vegetable oils and hydrogen gas into a variety of products. Some of these products do not have an inherent value, such as produced water and carbon dioxide. Valuable primary products include LPG, naphtha, renewable jet fuel and renewable diesel [9, 23, 24]. Besides these fuel products, Camelina and Carinata produce protein meal from the solvent extraction process, which can be traded in the animal feed market [9]. Jatropha meal, on the other hand, is toxic and cannot be commercialized [53]. However, biochar and shell ash, which are also tradable, are produced from Jatropha processing [22]. Table 7 lists valuable co-products generated by each evaluated feedstock and the respective unit prices used to calculate the total gross income of each facility.

It was assumed that fuel products produced from the HDO process meet the ASTM specifications for blending with or replacing their petroleum counterparts and so can be sold at an equivalent price. Fuel prices were taken from various references [17, 47–49]. The price for Camelina and Carinata meal was based on the price of canola meal, since they are new to the market [9, 50]. Prices for biochar were taken from another study [51]. Since shell ash prices in Australia were not available in the public domain, diammonium phosphate (DAP) fertilizer prices were used as a substitute [52]. All prices in Australian dollars were converted to US dollars (Table 5).

**Table 5:** Valuable co-products

	Unit prices (US$)
Camelina and Carinata	
LPG	0.62/L
Naphtha	0.99/L
Jet fuel	0.40/L
Diesel	0.98/L
Protein meal	0.35/kg
Jatropha	
LPG	0.62/L
Naphtha	0.99/L
Jet fuel	0.40/L
Diesel	0.98/L
Biochar	1.53/L
Shell ash	0.34/kg

Produced quantities of each fuel product were determined from the mass balance presented in Table 2. Quantities of Camelina and Carinata meal were dependent upon the product profile. For each 100 MLY of liquid fuel produced, 145,180 tonnes of Camelina meal are produced, compared to 97,450 tonnes of Carinata meal [9]. Biochar and shell ash were assumed to be produced in the same amounts in both product profiles and correspond to 26 and 251 kg/tonne of oil, respectively [22].

### Risk analysis

To better reflect the reality of the market and investigate to what extent the inputs’ uncertainties can influence the financial outputs of the projects being analysed, variations related to the independent variables described in Table 6 were identified and modelled using the @Risk software from Palisade Corporation, which uses Monte Carlo simulations to vary all the parameters simultaneously and present the aggregate impact on the techno-economic metrics.

**Table 6:** Variations of independent variables

Variables	(−)	Mean	(+)
CAPEX for HRD	177 MM	253 MM	329 MM
CAPEX for HRJ	296 MM	422 MM	549 MM
Camelina oil content (%)	30	35	40
Carinata oil content (%)	37	44	51
Jatropha oil content (%)	30	33	40
Camelina price	US$0.29/kg	US$0.32/kg	US$0.40/kg
Carinata price	US$0.33/kg	US$0.36/kg	US$0.45/kg
Jatropha price	US$0.23/kg	US$0.25/kg	US$0.31/kg
Hydrogen gas price	US$1.10/kg	US$1.21/kg	US$2.00/kg
Natural gas price	US$1.61/GJ	US$3.05/GJ	US$5.67/GJ
Electricity price	US$0.17/kWh	US$0.21/kWh	US$0.27/kWh
Water price	US$0.74/kL	US$1.62/kL	US$2.04/kL
LPG price	US$0.24/L	US$0.62/L	US$0.94/L
Naphtha price	US$0.38/L	US$0.99/L	US$1.49/L
Jet fuel price	US$0.16/L	US$0.40/L	US$0.60/L
Diesel price	US$0.38/L	US$0.98/L	US$1.48/L
Protein meal price	US$0.32/kg	US$0.35/kg	US$0.43/kg
Biochar price	US$1.38/L	US$1.53/L	US$1.88/L
Shell ash price	US$0.31/kg	US$0.34/kg	US$0.42/kg

The variations were based on percentages reported in previous literature and on fluctuations observed in the last 5 years [9, 22, 44–46, 54]. Variations in the prices of fuel products (LPG, naphtha, jet fuel and diesel) were assumed to be proportional to the variations in the price of crude oil, which is consistent with historical data for these products [9].

A Pert probability distribution, which uses three points estimate, was used for all the parameters. A convergence test was run on the P99 of the NPV, with a tolerance of ± 2% and a confidence of 95%, resulting in 47,000 iterations. This means that simulations should always be run with 47,000 iterations or more to produce similar results. By conservative means, simulations using 100,000 iterations were performed.

## Results and discussion

### Deterministic economic analysis

#### Plants maximizing the production of hydroprocessed renewable diesel

The economic viability of the hypothetical facility maximizing the production of renewable diesel was first evaluated using a deterministic approach. The variables were fixed at the values stated in Table 7. Variable OPEX and total revenue were calculated based on a plant operating in accordance with the fixed feed rate. The percentage that each co-product represented in the total revenue was also presented.

**Table 7:** Base case for production facilities targeting HRD and HRJ

	HRD			HRJ		
	Camelina	Carinata	Jatropha	Camelina	Carinata	Jatropha
Feedstock price (US$/tonne of seed)	323	356	254	323	356	254
Feedstock price (US$/tonne of oil)	923	809	770	923	809	770
CAPEX (US$MM)	253	253	253	422	422	422
OPEX (US$MM)	358	316	300	372	329	313
Total revenue (US$MM)	447	383	294	376	311	206
Revenue breakdown (%)						
LPG	3	3	4	5	5	8
Naphtha	1	2	2	11	15	9
HRJ	4	4	6	19	23	47
HRD	49	57	74	12	14	17
Protein meal	43	34	–	54	44	–
Biochar	–	–	4	–	–	6
Shell ash	–	–	10	–	–	14

The risk-adjusted discount rate to bring the series of cash flows to an NPV of zero was calculated at 9.71%. Therefore, IRRs higher than this indicate positive NPVs and attractive investment opportunities. Camelina and Carinata facilities returned IRRs of 25 and 18% and NPVs of 353 and 185 US$MM, respectively. Jatropha facility had no IRR and NPV of − 387 US$MM. Only the Camelina and Carinata scenarios exceeded the 9.71% IRR, and thus had positive NPVs. The payback time for the Camelina scenario was 5.5 years, against 8.3 years for the Carinata scenario.

With the highest IRR and NPV, the Camelina plant offered the shortest payback time, and, consequently the best business case, despite having the highest operating costs. Camelina has lower oil content than Carinata and, because of this fact, requires more feedstock to produce the same quantity of extracted oil for conversion to HRD. However, Camelina generates substantially higher revenue due to the larger production of protein meal. It is important to note that the revenues of both Camelina and Carinata scenarios were strongly dependent on the sale of meal; which represented 43% of the revenues for Camelina and 34% of the revenues for Carinata.

The lower revenue from the protein meal for Carinata justified the lower IRR and NPV compared to Camelina. The Jatropha facility simultaneously exhibited the lowest operating costs and the smallest revenue. It can be seen from Table 7 that Jatropha was the cheapest feedstock among the three biomass materials which were analysed. The price of Jatropha seed was approximately 21 and 29% lower than the Camelina and Carinata seed prices, respectively. However, the price reduction dropped to 16 and 0.05% when the oil prices were compared against each other. This disparity was due to the differences in the oil content of each feedstock. When it comes to the total revenue generated by the sale of the co-products, it was not sufficient to cover the operating costs. This calculation explained the resultant negative value of NPV and non-existent IRR. The Jatropha meal is toxic and cannot be traded [53]. In addition to the fuel products, Jatropha produced biochar and shell ash, which can be commercialized, but the quantity produced is not enough to significantly influence the total revenue value, in contrast to the situation with protein meal generated by both Camelina and Carinata.

Additional scenarios considering shortages of feedstock were also analysed. A production facility converting Camelina and operating at 50% of its capacity would reduce the NPV to − 50 US$MM and the IRR to 7%, thus becoming economically unattractive. On the other hand, NPV and IRR of the same plant operating at 75% of its capacity would be US$151 MM and 17%, respectively. For the Carinata scenario, the NPV and IRR would be − 134 US$MM and 2% for a 50% operating capacity plant, and US$25 MM and 11% for a 75% operating capacity plant. Therefore, a 25% reduction in production would not affect the economic viability of the Camelina and Carinata plants. On the contrary, an increase in the capacity by 25% would return NPVs and IRRs of US$554 MM and 33% for Camelina, and US$345 MM and 25% for Carinata.

Research conducted by Chu et al. [9] also culminated in positive NPVs and attractive IRRs for HRD scenarios processing Camelina and Carinata. They returned NPVs of 195 and 121 US$MM, and IRRs of 28 and 23%, respectively. The noted differences between the results of this project and the ones reported by Chu et al. should be ascribed to the distinct discounted cash flow models, and prices of both inputs and outputs, which in the compared study were based on the Canadian context. An economic analysis of jet fuel produced from Jatropha, described in the literature [22], evaluated only the HRJ product profile, and determined only the minimum selling price of jet fuel. Results for financial parameters were not made available.

#### Plants maximizing the production of hydroprocessed renewable jet fuel

The comparative variables of the hypothetical production facilities maximizing the production of renewable jet fuel are presented in Table 6. The Camelina, Carinata and Jatropha scenarios returned NPVs of − 493, − 717 and − 1.627 US$MM, respectively. IRRs were non-existent in all the cases. With these latter results, all scenarios for HRJ were unattractive. The principal disadvantages of the HRJ projects over the HRD ones are related to three main aspects: the necessity of additional process unit operations; differences in the product yields; and relative selling prices of jet fuel and diesel.

When maximizing the production of jet fuel, the use of isomerization unit was necessary [9, 23, 24]. This situation not only made the CAPEX 67% more costly, but also increased the fixed OPEX, since it was based on the total project investment. Furthermore, as the name suggested, HRJ projects increased the production of jet fuel, while concomitantly decreasing the production of diesel. However, the selling price of jet fuel in Australia was approximately 60% lower than the selling price of diesel. For this latter reason, the total revenue generated in the HRJ scenarios reduced considerably, representing reductions of 16, 19 and 30% for the Camelina, Carinata and Jatropha scenarios, respectively.

Total revenues generated by Carinata and Jatropha facilities were not even sufficient to cover the operating expenses. For the Camelina facility, it would be necessary to increase capacity by 169%, so that it could return NPV equal to zero and IRR of 9.71%.

For the HRJ facilities, the results reported by Chu et al. [9] presented bigger distortions. Camelina and Carinata returned NPVs of 35 and − 29 US$MM, respectively. In this case, it is important to highlight that the selling price of kerosene in Canada, as stated, was 80% higher than the selling price of the same product in Australia. Therefore, as expected, Canadian HRJ scenarios are less pessimistic than Australian.

#### Break-even analysis

Break-even analysis was performed to determine the minimum selling prices of the fuel products and the maximum cost of the feedstock that each model could tolerate to return NPV equal to zero and IRR of 9.71%. The main assumptions used while calculating the break-even prices of the fuel products were that protein meals, biochar and shell ash prices would be constant, and the prices of each type of fuel (LPG, naphtha, jet fuel and diesel) would change in the same proportion.

##### Plants maximizing the production of hydroprocessed renewable diesel

The break-even prices for LPG, naphtha, jet fuel and diesel produced from Camelina in an HRD plant were 0.50, 0.80, 0.32 and 0.79 US$/L, respectively; which represented a reduction of 19% relative to the current market prices in Australia, presented in Table 8. For the Carinata facility, the break-even fuel prices were 10% lower than the market prices: 0.56, 0.89, 0.36 and 0.88 US$/L for LPG, naphtha, jet fuel and diesel, respectively. On the other hand, for the Jatropha project the break-even prices were 19% higher than the current prices, reaching 0.74, 1.18, 0.48 and 1.16 US$/L for LPG, naphtha, jet fuel and diesel, respectively. The average market prices and the calculated break-even fuel prices for the hypothetical production facilities maximizing the production of renewable diesel are presented for comparison in Table 8.

**Table 8:** Break-even fuel prices for production facilities targeting HRD

	LPG (US$/L)	Naphtha (US$/L)	Jet fuel (US$/L)	Diesel (US$/L)
Market prices	0.62	0.99	0.40	0.98
Camelina	0.50	0.80	0.32	0.79
Carinata	0.56	0.89	0.36	0.88
Jatropha	0.74	1.18	0.48	1.16

Regarding the break-even prices for feedstocks, the financial models estimated that HRD facilities processing Camelina and Carinata could tolerate increases in feedstock prices by 16 and 10%, respectively. This meant that, keeping all the other variables at the fixed values, Camelina and Carinata prices could rise from 923 to 1070 US$/tonne of oil, and from 809 to 886 US$/tonne of oil, respectively; then the NPV would be zero and the IRR of 9.71% would be achieved. However, in the case of a facility processing Jatropha, the feedstock price would have to be reduced from 770 to 624 US$/tonne of oil to reach the break-even point.

##### Plants maximizing the production of hydroprocessed renewable jet fuel

As all the scenarios maximizing the production of jet fuel returned negative NPVs, the selling prices of the fuel products would have to increase or the cost of the feedstock would have to decrease to achieve the break-even point. The break-even prices calculated for LPG, naphtha, jet fuel and diesel produced from Camelina were 37% higher than the average market prices: 0.85, 1.36, 0.55 and 1.34 US$/L, respectively. For the Carinata scenario, the minimum selling prices of the fuel products to return NPV equal to zero and IRR of 9.71% were 0.92, 1.48, 0.60 and 1.46 US$/L for LPG, naphtha, jet fuel and diesel, respectively; which represented an increase of 49% relative to the current market prices. It was the Jatropha project, however, which required the largest increase. Fuel products from this feedstock would have to be sold for a price 106% higher than the actual prices to reach the break-even point. In this case, break-even prices for LPG, naphtha, jet fuel and diesel were 1.28, 2.04, 0.82 and 2.02 US$/L, respectively. The average market prices and the calculated break-even fuel prices for the hypothetical production facilities maximizing the production of renewable jet fuel are presented for comparison in Table 9.

**Table 9:** Break-even fuel prices for production facilities targeting HRJ

	LPG (US$/L)	Naphtha (US$/L)	Jet fuel (US$/L)	Diesel (US$/L)
Market prices	0.62	0.99	0.40	0.98
Camelina	0.85	1.36	0.55	1.34
Carinata	0.92	1.48	0.60	1.46
Jatropha	1.28	2.04	0.82	2.02

In regard to the break-even prices for feedstocks, the financial models demonstrated that HRJ facilities would require reductions of 21, 33 and 69% in the prices of Camelina, Carinata and Jatropha, respectively. In other words, to achieve NPV equal to zero and IRR equal to 9.71%, the price of Camelina would have to decrease from 923 to 725 US$/tonne of oil, the price of Carinata would have to decline from 809 to 545 US$/tonne of oil and the price of Jatropha would have to fall from 770 to 235 US$/tonne of oil, while keeping all the other variables at the fixed values.

Previous articles [9, 22] reported break-even prices of 0.69, 0.74 and 1.43 US$/L of jet fuel produced from Camelina, Carinata and Jatropha, respectively. Jatropha fuel accounts for the biggest variance between previous and current results. This might be attributed to significant differences in plant assumptions, such as production capacity.

### Stochastic analysis

To examine the riskiness of investments in the aviation biofuels sector, uncertainties related to key variables were considered and modelled into @Risk software, using Pert probability distributions. These variables include: capital expenses, oil content and prices of feedstocks, prices of hydrogen gas and utilities and prices of co-products. All of them were assumed to be independent of each other.

#### Plants maximizing the production of hydroprocessed renewable diesel

**Fig. 2:**
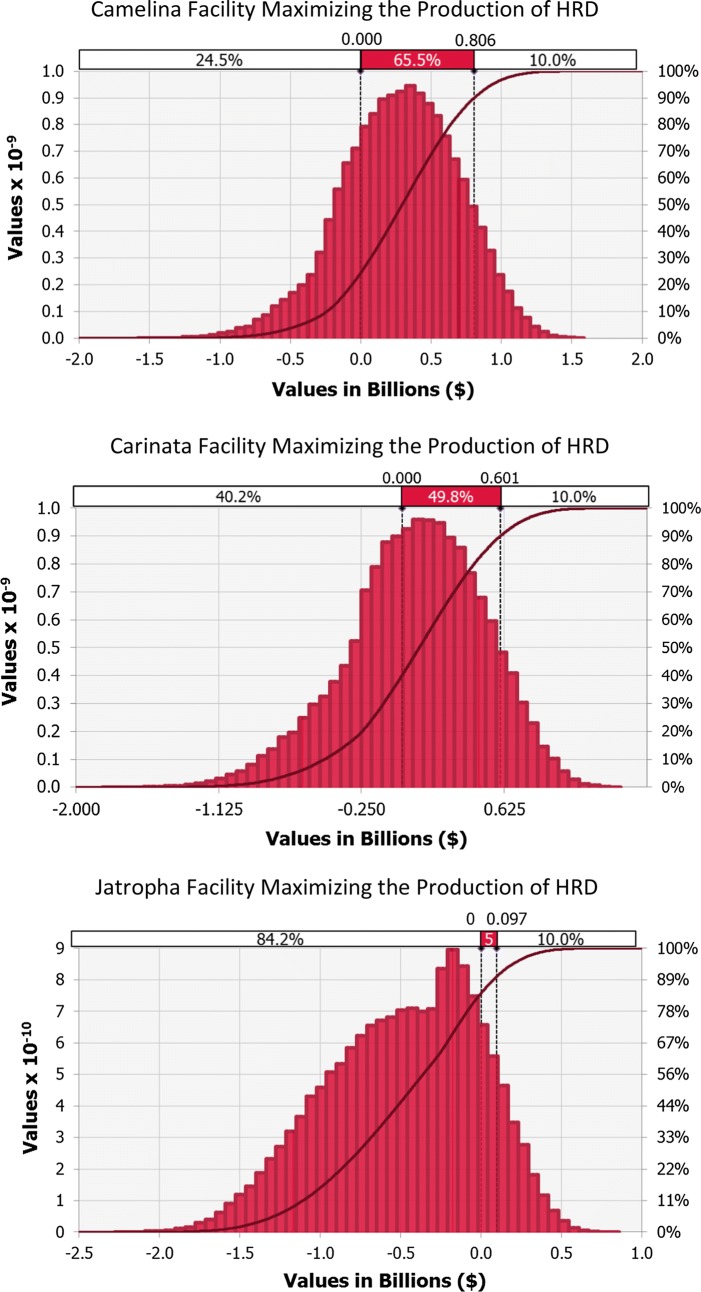
Probability distributions for the facilities targeting HRD

Monte Carlo probability distributions in the NPV of the Camelina, Carinata and Jatropha plants maximizing the production of hydroprocessed renewable diesel are presented in Fig. 2. The solid shaded areas on the graphs are the probabilities that range from NPV zero to P90. Therefore, the probabilities of having positive NPVs and, consequently, IRRs higher than 9.71% are 75.5, 59.8 and 15.8% for Camelina, Carinata and Jatropha, respectively. As well as in the deterministic analysis, the best scenario is Camelina’s, which has 90% chance of achieving an NPV of US$805 MM, a value 218% higher than the TPI. It is followed by Carinata, the second most attractive business case. Despite the considerable probability of loss of 40.2%, the P90 for the Carinata plant is US$602 MM, which is 137% higher than the TPI. In regard to the facility processing Jatropha, although it has been revealed as non-economically viable under a deterministic approach, some chance of having a positive NPV has been shown in the stochastic analysis. However, with 84.2% probability of loss it is highly risky. Its P90 is US$95 MM, which represents only 16% of the P90 returned by Carinata, and 12% of the P90 returned by Camelina.

A sensitivity analysis performed for the three scenarios revealed that the contribution of the key variables to the variance in the NPV was very similar in all the facilities, as shown in Figures S1 and S2 in Additional file 1: B. The price of diesel had the greatest effect on the output mean and was positively correlated with it. It was followed by the feedstocks’ price and oil content, which occupied the second and third positions. The prices of feedstocks were negatively correlated with NPVs, while the correlation of the oil content was positive. For the Camelina and Carinata scenarios, the price of protein meal was the fourth most significant parameter, positively related to NPVs, whereas Jatropha had the same position occupied by the CAPEX, negatively correlated instead. Capital expenses were the fifth most influential parameter for Camelina and Carinata.

In general, other variables (prices of other co-products, utilities and hydrogen gas) had relatively low influence on the NPV of the three plants.

#### Plants maximizing the production of hydroprocessed renewable jet fuel

**Fig. 3:**
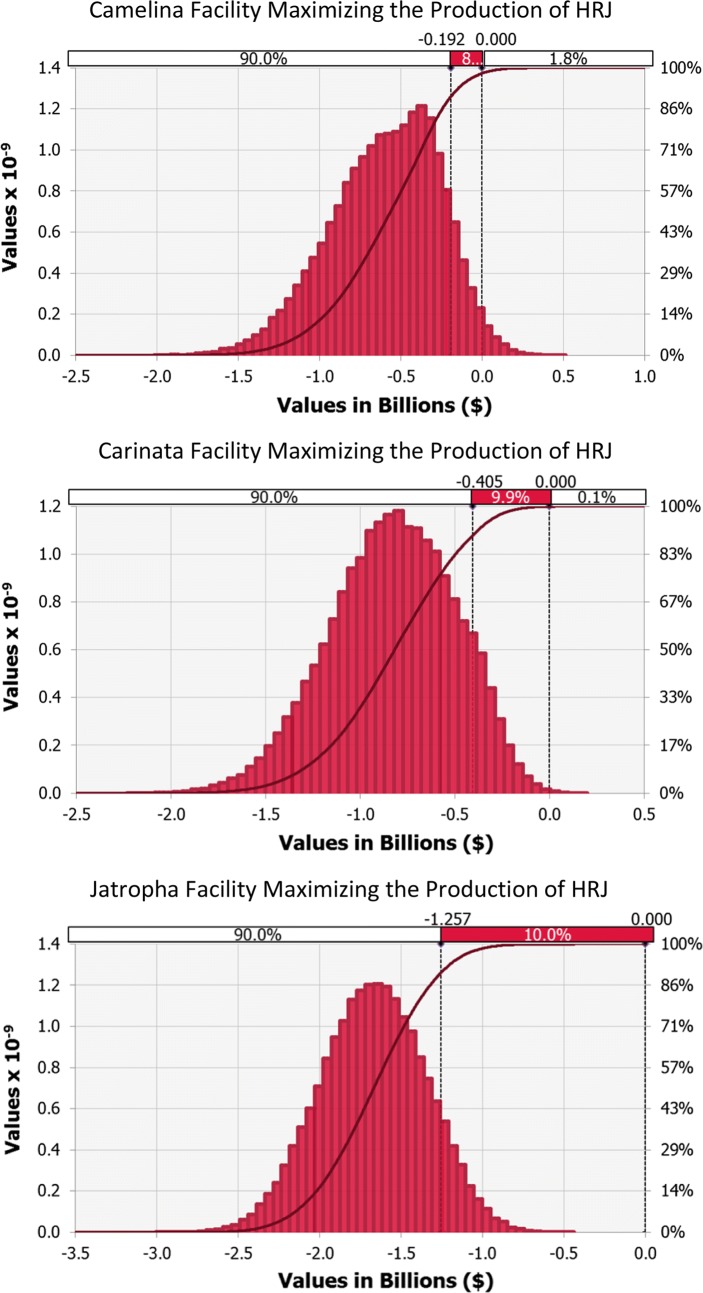
Probability distributions for the facilities targeting HRJ

None of the facilities maximizing the production of hydroprocessed renewable jet fuel presented reasonable probabilities of having positive NPVs. On the contrary, extreme probabilities of loss of 98.2% for Camelina, 99.9% for Carinata and 100% for Jatropha were observed, as presented in Fig. 3. All three scenarios presented negative P90s: −192 US$MM for Camelina, − 405 US$MM for Carinata and − 1257 US$MM for Jatropha. Therefore, these projects were not potentially profitable under neither deterministic nor stochastic analyses.

In regard to the sensitivity analysis performed for the HRJ scenarios (see Additional file 1: B), it was also possible to note similar effects of the key variables on the NPV of each facility. In these cases, the first three positions were occupied by the oil content and prices of feedstocks and price of kerosene. The order of significance, however, differed according to the feedstock. For Camelina, the parameter that greatest impacted the output mean was its price, followed by the oil content of its seed and the selling price of kerosene. For Carinata, the oil content of its seed was the most influential parameter, followed by its price and the selling price of kerosene. For the Jatropha scenario, the price of kerosene was the most significant variable, followed by its price and the oil content of its seed. In all the scenarios, prices of feedstocks were negatively correlated with NPVs, while feedstocks’ oil content and price of kerosene were positively correlated with them. Other variables such as price of other co-products and CAPEX contributed approximately with ± 10% or less to the variance in NPVs.

Chu et al. [9] reported probabilities of having positive NPVs of 29 and 18% for Camelina and Carinata plants, respectively. No article examining the robustness of the financial performance of plants processing Jatropha was found in the literature. The differences between the probabilities determined in this paper and in the research being compared are very closely related to the selling price of jet fuel. The facilities modelled by Chu et al. were designed to operate in Canada, where the selling price of kerosene was US$0.72/L in 2015. This price is 80% higher than the average price of jet fuel in Australia. The fact that this variable is among those which most contributes to the variance in the financial outputs, as demonstrated in the sensitivity analysis, explains the disparity in the results.

### Policy analysis

It was difficult to know exactly at what point in the probability distributions investors would be willing to sponsor a project. Bittner et al. [3] believed that firms would place a bid at 20 or 30% of probability of loss. For policy analysis purposes, this project assumed the maximum probability of loss proposed by them, 30%. Considering this, the Camelina facility maximizing the production of renewable diesel would be the only scenario within the acceptable risk limits. All other plants would require economic subsidies from the government to become attractive for investments. To examine the magnitude of these contributions, this project studied two main categories of stimulus based on the results of the sensitivity analyses. Thus, the first study focused on increasing revenue by raising the prices of output fuels, while the second exercise focused on reducing costs from feedstocks.

In the first scenario, incentives of 0.06 and 0.28 US$/L of biofuel produced would be necessary to reduce the probability of loss to approximately 30% for Carinata and Jatropha facilities maximizing the production of HRD, respectively. On the other hand, Camelina, Carinata and Jatropha facilities targeting the production of HRJ would demand incentives of 0.31, 0.39 and 0.61 US$/L of biofuel produced to reach the acceptable risk limits.

In the second scenario, Carinata and Jatropha facilities maximizing the production of HRD would require incentives of 0.02, 0.08 US$/kg of oilseed purchased, respectively, to decrease the probability of loss to approximately 30%. Camelina, Carinata and Jatropha plants maximizing the production of HRJ would need incentives of 0.10, 0.15 and 0.20 US$/kg of oilseed purchased, respectively, to achieve the acceptable risk limits.

The Camelina, Carinata and Jatropha plants operating in accordance with the feed rate of 39 tonnes of oil per hour would require approximately 111, 89 and 118 tonnes of seed per hour, respectively, taking into account the differences in their oil content. Based on the product yields, 903 L of biofuel would be produced, on average, per tonne of oil processed. Considering the same processing rate of 39 tonnes of oil per hour, both categories of incentives would represent very similar costs to the government.

## Conclusions

Under a deterministic approach, only the Camelina and Carinata facilities maximizing the production of renewable diesel are attractive investment opportunities. They returned, respectively, NPVs of 353 and 185 US$MM, and IRRs of 25 and 18%. All other scenarios had negative NPVs and no IRR. It was noted that the economically viable plants are highly dependent on the sale of protein meal in addition to the fuel products, since it represented 43% of the total revenue generated by Camelina and 34% of the revenue for Carinata. Three main aspects are relevant to explain the disadvantages of HRJ over HRD projects: necessity of additional processing, differences in product yields and relative selling prices of jet fuel and diesel. This combination simultaneously increases costs and decreases revenue.

Considering the volatility of the market, the probabilities that the NPV would be positive and the IRR would be higher than 9.71% were 75.5, 59.8 and 15.8% for the Camelina, Carinata and Jatropha plants targeting the production of HRD, respectively. Extremely high probabilities of loss for all the facilities maximizing the production of HRJ were found: 98.2, 99.9 and 100% for the Camelina, Carinata and Jatropha scenarios, respectively. Assuming that investors would only be willing to sponsor projects with probabilities of loss of 30% maximum, only the Camelina project targeting HRD would be an attractive business case. All other options would require the implementation of subsidy policies to increase profits, consequently enhancing their attractiveness.

To conclude, the results indicate that Jatropha is a disadvantageous option for both product profiles that were assessed, and under both deterministic and stochastic conditions. Camelina, on the other hand, is the best scenario. A facility processing this feedstock and targeting HRD is financially viable under deterministic and stochastic conditions. When maximizing the production of jet fuel, the Camelina project requires incentives to reach the acceptable risk limits, but they are lower compared to the contributions required by the Carinata and Jatropha facilities. Therefore, Carinata would be the second best option, but it would require subsidies for the deployment of both product profiles, HRD and HRJ.

Although the developed model was sufficient for conducting scenario analysis, there is potential in future studies to refine the model even more. For example, changes in price escalation and inflation rates, as well as process input, outputs and utility costs are obvious choices for sensitivity analysis. More complicated risk analysis with forecasting and predictive simulations could also be used with software programs such as NeuralTools from Palisade Corporation and Crystal Ball from Oracle. Another improvement to the model might include greater focus on break-even analysis that considers future price trend and uncertainties.

## Additional file

**Additional file 1.** Capital expense assumptions & stochastic modelling data.
