# Supplementary material for: Stochastic techno-economic analysis of the production of aviation biofuel from oilseeds
Source: Biotechnol Biofuels. 2018 Jun 8;11:161. doi: 10.1186/s13068-018-1158-0 (PMC5992646; doi:10.1186/s13068-018-1158-0)
Supplement: Supplementary file 1 — Additional file 1. Capital expense assumptions & stochastic modelling data. [file 13068_2018_1158_MOESM1_ESM.docx]

**Additional Section**

**A: Capital Expense Assumptions**

Table S1: Capital expenses

| Total Equipment Installed Cost(TEIC) | Oil Extraction |
| --- | --- |
|  | HDO |
|  | Product Separation |
|  | Power Island |
|  | Pumps, Tanks, Heat Exchangers |
|  | Other Direct Cost: Site Development |
| Total Direct Cost (TDC) | TEIC + Other Direct Cost |
| Total Indirect Cost (TIC) | Field Expenses |
|  | Home Office & Construction Fee |
|  | Project Contingency |
|  | Other costs (permitting and construction insurance) |
| Total Capital Investment (TCI) | TDC + TIC |
| Working Capital (WC) | 10% of TCI |
| Total Project Investment (TPI) | TCI + WC |

**B: Supplementary Results of Stochastic Analyses**

| Camelina Facility Maximizing the Production of HRD |
| --- |
| 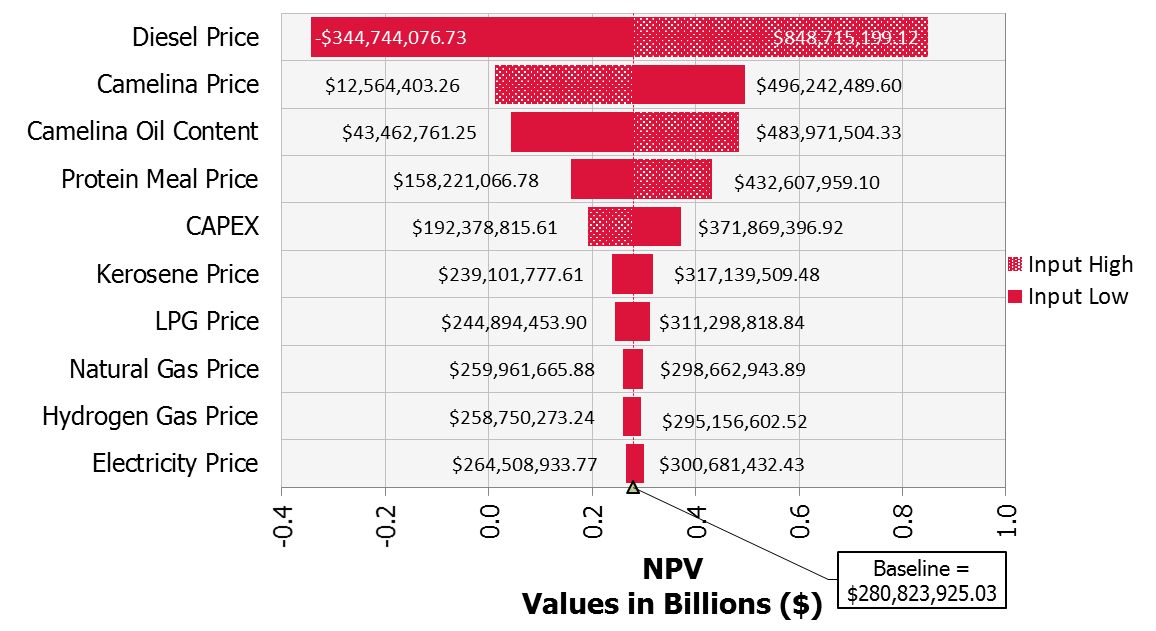 |
| Carinata Facility Maximizing the Production of HRD |
| 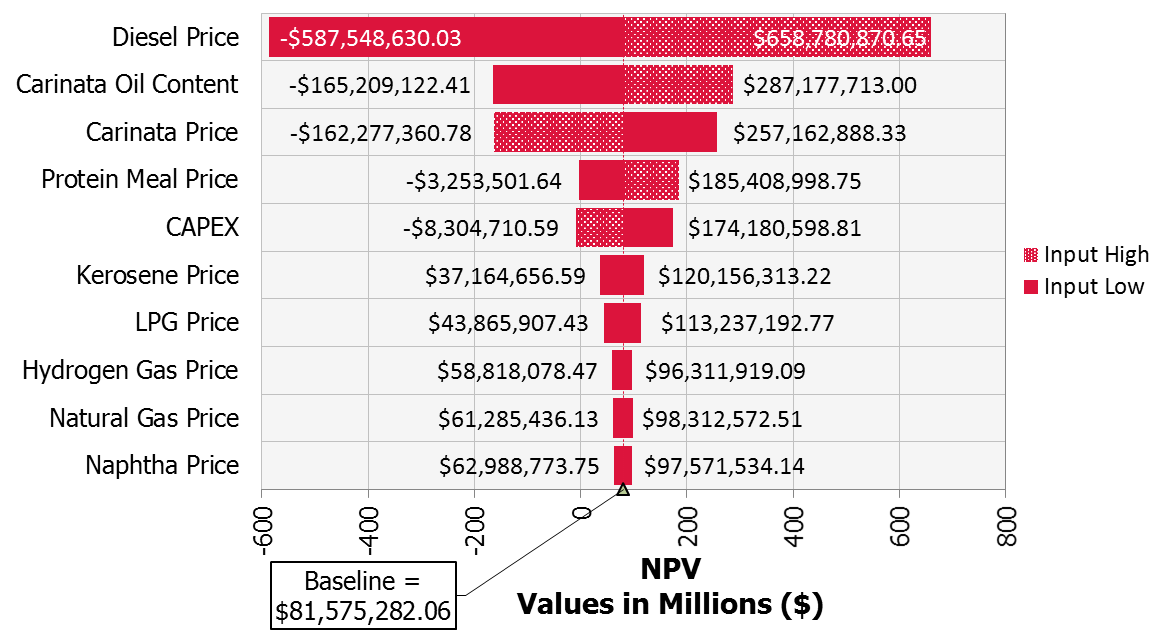 |
| Jatropha Facility Maximizing the Production of HRD |
| 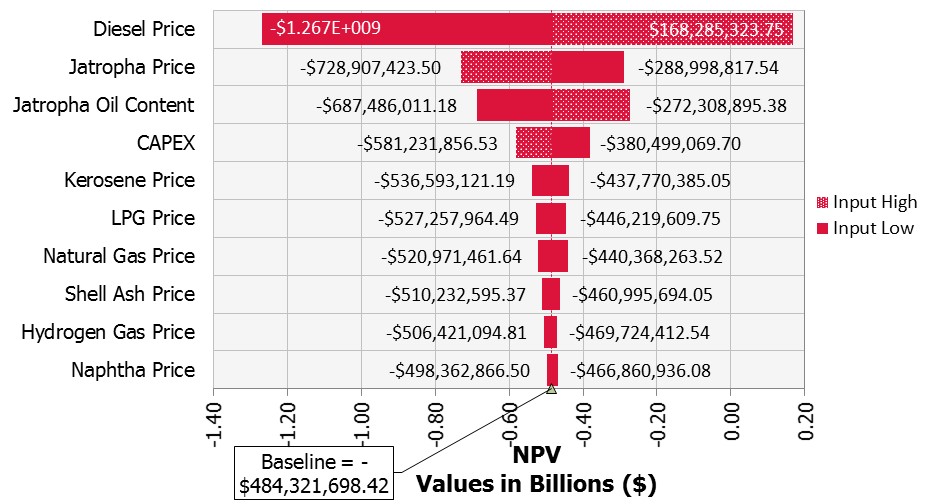 |

Figure S1: Inputs of the facilities targeting HRD ranked by effect on the output mean

| Camelina Facility Maximizing the Production of HRD |
| --- |
| 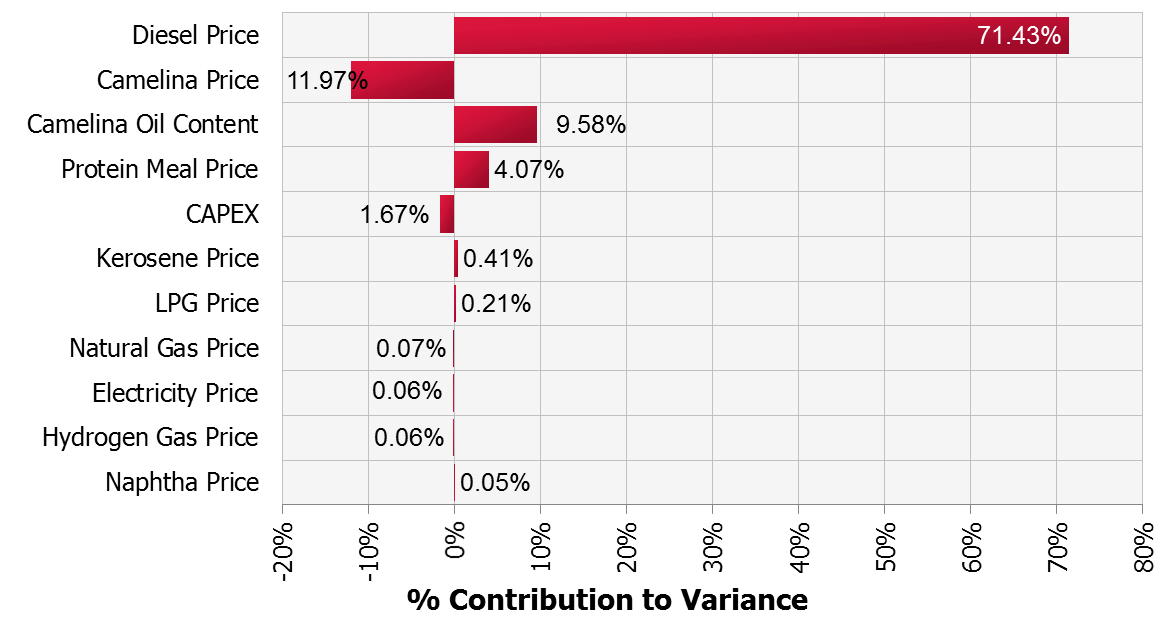 |
| Carinata Facility Maximizing the Production of HRD |
| 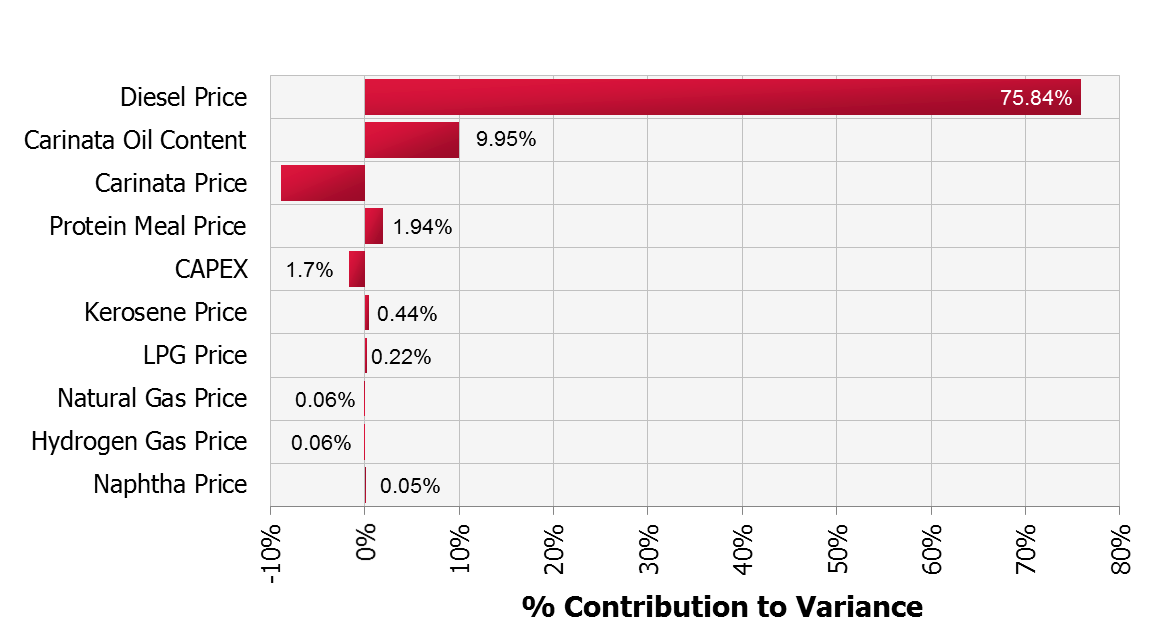 |
| Jatropha Facility Maximizing the Production of HRD |
| 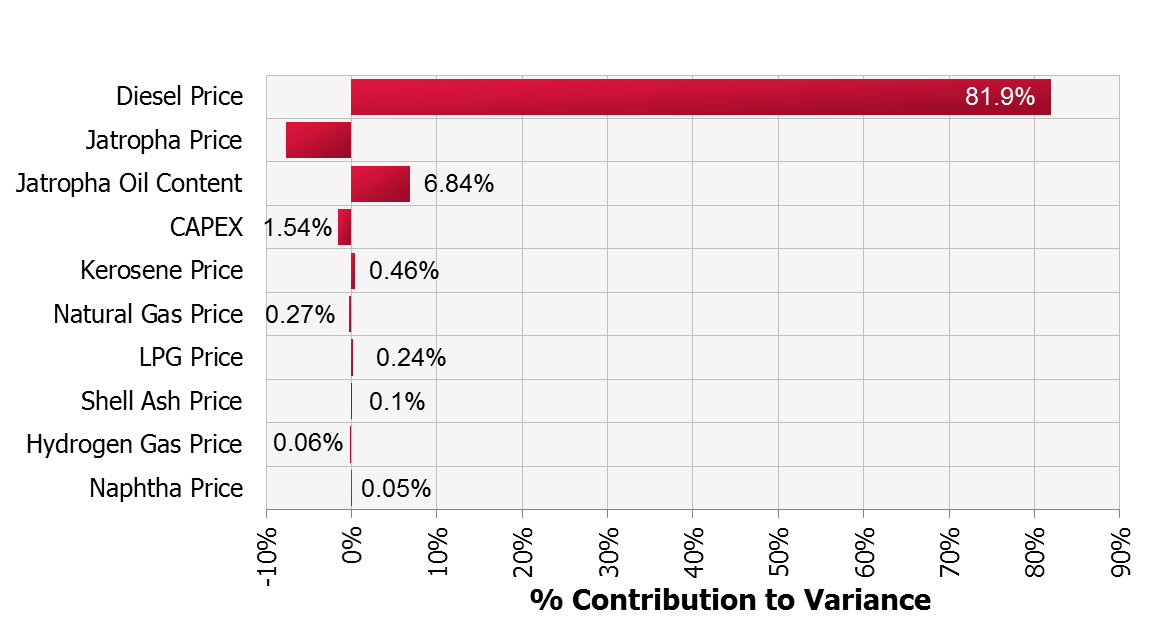 |

Figure S2: Contribution to variance in the NPVs of the facilities targeting HRD

| Camelina Facility Maximizing the Production of HRJ |
| --- |
| 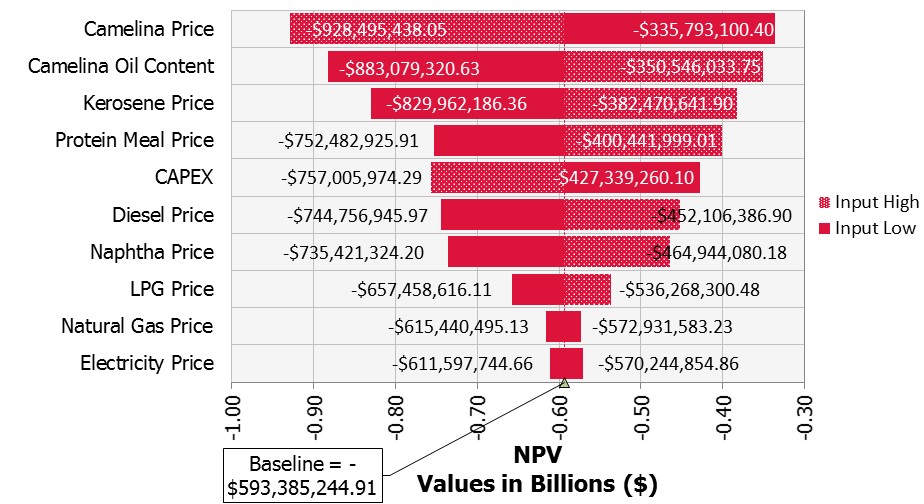 |
| Carinata Facility Maximizing the Production of HRJ |
| 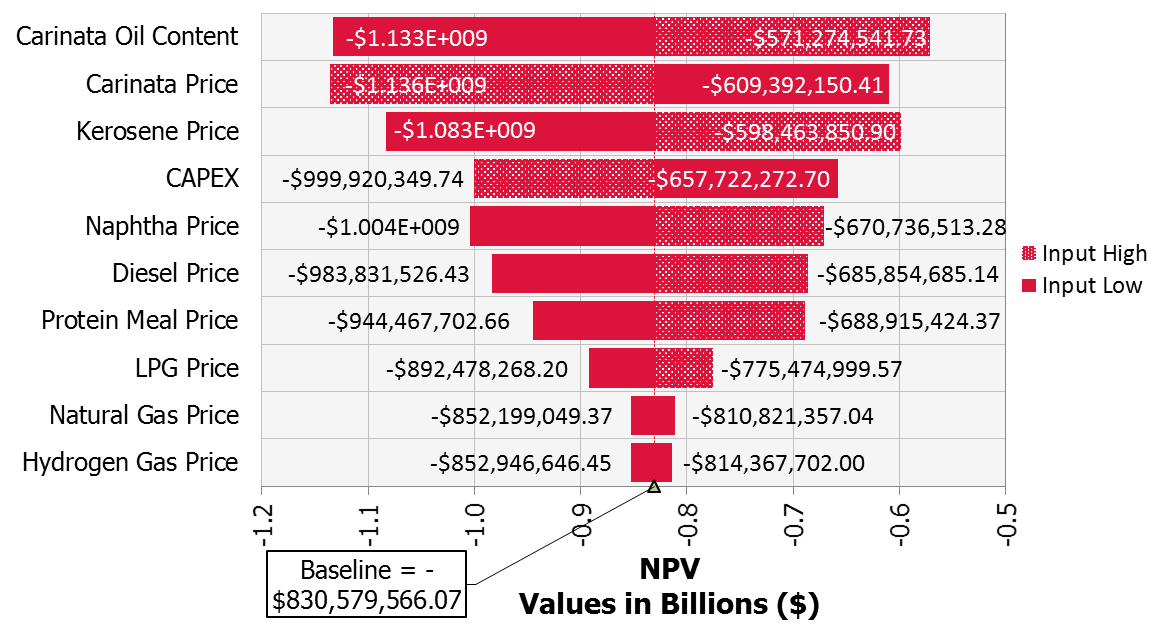 |
| Jatropha Facility Maximizing the Production of HRJ |
| 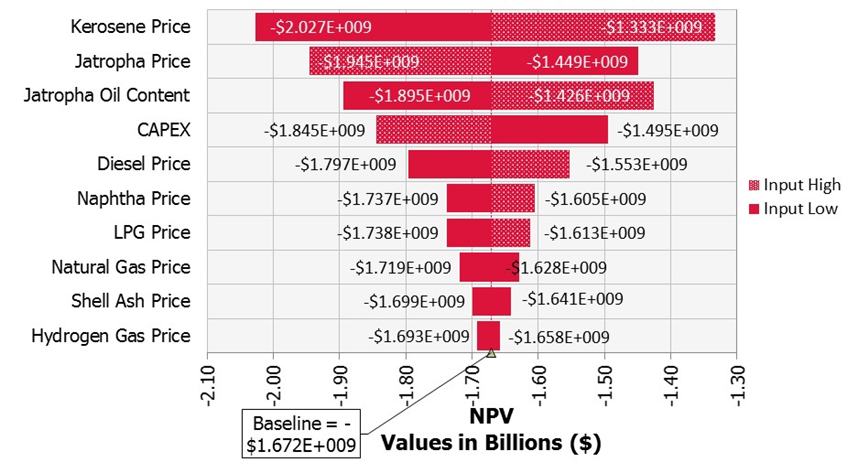 |

Figure S3: Inputs of the facilities targeting HRJ ranked by effect on the output mean

| Camelina Facility Maximizing the Production of HRJ |
| --- |
| 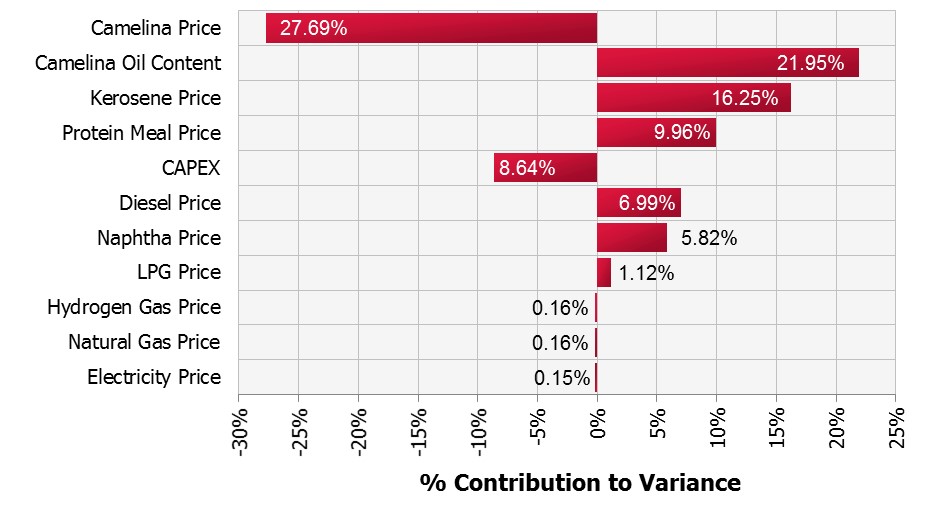 |
| Carinata Facility Maximizing the Production of HRJ |
| 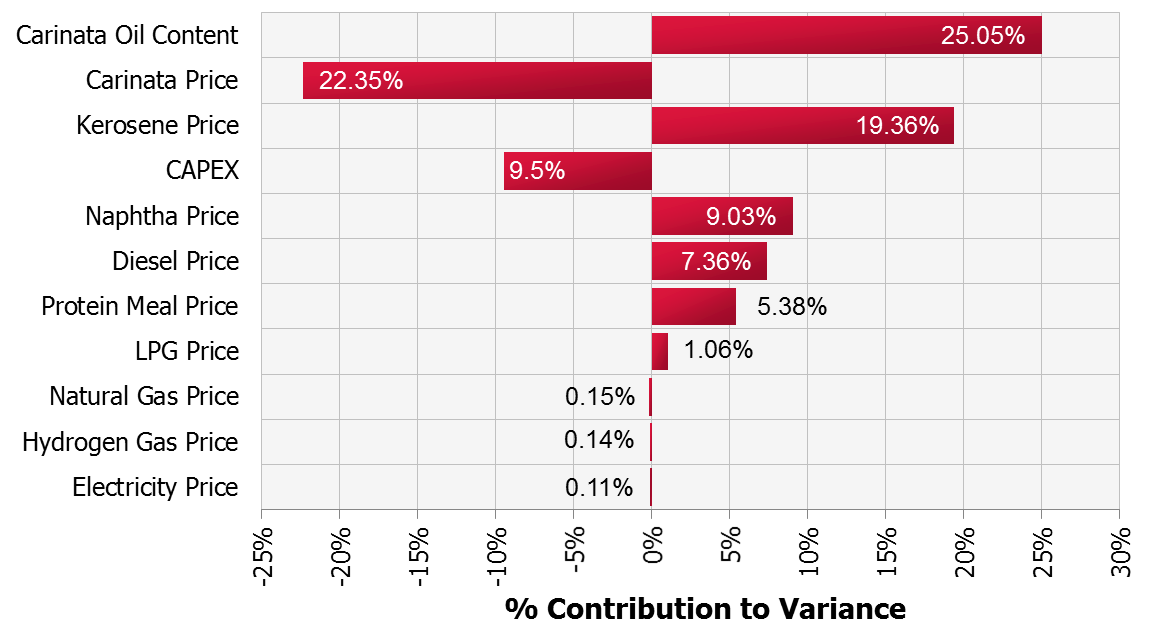 |
| Jatropha Facility Maximizing the Production of HRJ |
| 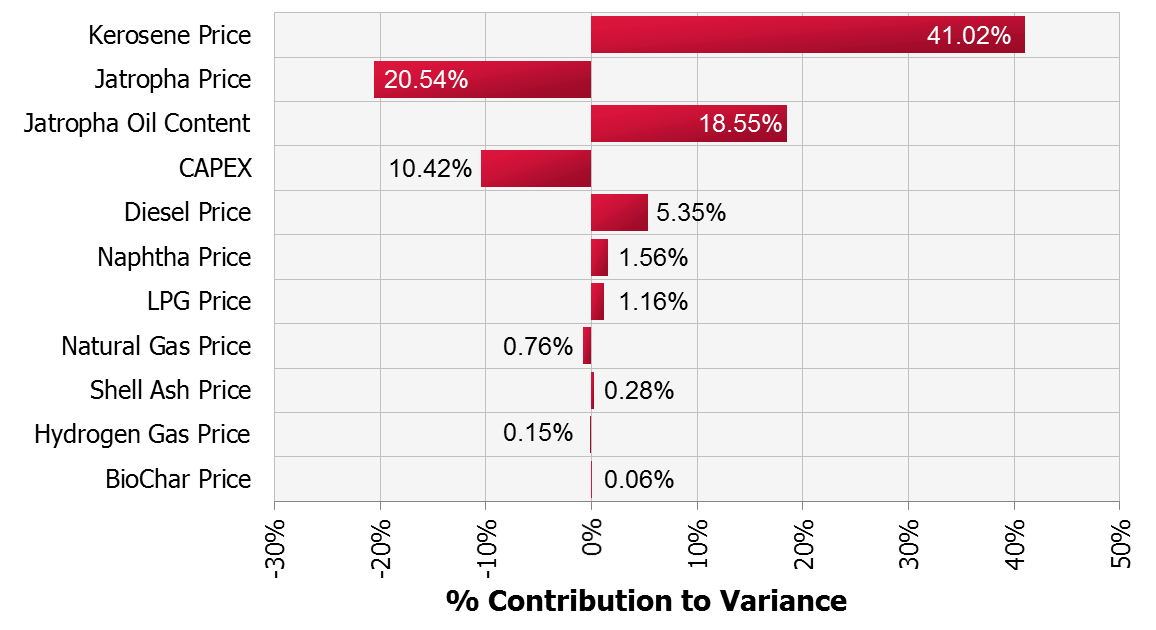 |

Figure S4: Contribution to variance in the NPVs of the facilities targeting HRJ

**C: Weighted Average Cost of Capital Formulas and Calculations**

The formula used to calculate the WACC is presented in Equation 1.

Equation 1: $WACC= \frac{E}{E+D}r_{E}+\frac{D}{E+D}r_{D}(1-Tc)$

Where: *E* = market value of the firm’s equity; *D* = market value of the firm’s debt; *Tc* = firm’s corporate tax rate; *r_E_* = firm’s cost of equity; *r_D_* = firm’s cost of debt.

The firm’s cost of equity was calculated by means of Equation 2:

Equation 2: $r_{E}=r_{f}+ \beta\left[ E\left( r_{M} \right)- r_{f} \right]$

Where: $r_{f}$ = the market risk-free rate of interest; $E\left( r_{M} \right)$ = the expected return on the market portfolio; $\beta$ = a firm-specific risk measure.

Beta, in its turn, was calculated using Equation 3:

Equation 3: $\beta= \frac{\left( r_{stock}-r_{f} \right)}{\left( r_{M}-r_{f} \right)}$

Where: $r_{stock}$ = stock rate of return; $r_{M}$ = market rate of return.
